# ERGA-BGE genome of *Leiobunum subalpinum* - a montane-subalpine harvestman species endemic to the eastern Alps

**DOI:** 10.12688/openreseurope.21990.1

**Published:** 2026-03-26

**Authors:** Peter Kozel, Aja Bončina, Elena Bužan, Astrid Böhne, Rita Monteiro, Rosa Fernández, Nuria Escudero, Marta Gut, Laura Aguilera, Francisco Câmara Ferreira, Fernando Cruz, Jèssica Gómez-Garrido, Tyler S. Alioto, Fergal Martin, Vianey Paola Barrera Enriquez, Leanne Haggerty, Chiara Bortoluzzi

**Affiliations:** 1 Faculty of Natural Sciences and Mathematics, Koroška cesta 160, University of Maribor, Maribor, 2000, Slovenia; 2 Karst Research Institute ZRC SAZU, Titov trg 2, Postojna, 6230, Slovenia; 3 Faculty of Mathematics, Natural Sciences and Information Technologies, Glagoljaška 8, University of Primorska, Koper, SI-6000, Slovenia; 4 Faculty of Environmental Protection, Trg mladosti 7, Velenje, 3320, Slovenia; 5 Leibniz Institute for the Analysis of Biodiversity Change, Adenauerallee 127, Museum Koenig Bonn, Bonn, 53113, Germany; 6 6 Metazoa Phylogenomics and Genome Evolution Lab, Passeig marítim de la Barceloneta 37-49., Institute for Evolutionary Biology (CSIC-UPF), Barcelona, 08003, Spain; 7 Centro Nacional de Análisis Genómico (CNAG), Baldiri Reixac 4, Barcelona, 08028, Spain; 8 Universitat de Barcelona (UB), Barcelona, 08028, Spain; 9 Wellcome Genome Campus, European Molecular Biology Laboratory, Hinxton, UK; 10 SIB Swiss Institute of Bioinformatics, Amphipôle, Quartier UNIL-Sorge,, Lausanne, 1015, Switzerland

**Keywords:** Leiobunum subalpinum, genome assembly, European Reference Genome Atlas, Biodiversity Genomic Europe, Earth Biogenome Project, Sclerosomatidae, Subalpiner Schwarzrückenkanker

## Abstract

*Leiobunum subalpinum* is a medium-sized, long-legged harvestman inhabiting montane to subalpine habitats in the eastern Austrian Alps and the neighbouring eastern Alps in Slovenia. The species is caldophilus and hygrophilus, occurring primarily in forested and rocky habitats, especially on siliceous rock faces on southern mountain slopes. As a result of its high susceptibility even to minor microclimatic variation, *L. subalpinum* may serve as an important indicator species of montane–subalpine ecosystem conditions. Moreover, as the species is restricted to a narrow geographical and altitudinal range, it represents an important regional biodiversity element and a relevant species for prioritizing the conservation of high-altitude habitats. The reference genome of *L. subalpinum* provides a crucial resource for uncovering the genetic differences between this species and the morphologically similar congener *L. rupestre.* The entirety of the genome sequence was assembled into 11 contiguous chromosomal pseudomolecules. This chromosome-level assembly encompasses 0.58 Gb, composed of 265 contigs and 44 scaffolds, with contig and scaffold N50 values of 5.2 Mb and 52.6 Mb, respectively.

## Introduction

*Leiobunum subalpinum* is a medium-sized, long-legged harvestman (leg span up to 14 cm) from the family *Sclerosomatidae* (subfamily *Leiobuninae*) ( Komposch, 1998) ( Figure 1). The species is distributed in the eastern Austrian Alps and the neighbouring eastern Alps in Slovenia, where its range is firmly nested within that of *L. rupestre* ( Komposch, 1998; Kozel et al., 2021; Martens & Schönhofer, 2016). *Leiobunum subalpinum* is a montane to subalpine species, with vertical distribution of 430–2200 m a.s.l. in Austria ( Komposch & Gruber, 1999; Martens & Schönhofer, 2016) and between 635–1425 m a.s.l. in Slovenia. The species prefers forested and rocky habitats, primarily siliceous rock faces on southern mountain slopes ( Komposch, 1998; Kozel et al., 2021). In some locations, *L. subalpinum* occurs in syntopy with *L. rupestre*, occupying microhabitats with slightly wider air temperature–relative humidity ecospace than *L. rupestre* during daytime rest ( Kozel et al., 2021). Adults are stenochronous from June to December, depending on altitude, juveniles are present from October to June and overwinter ( Komposch, 1998; Kozel et al., 2021). This species is considered most closely related to, and very similar to, *L. rupestre*, including the genital morphology; therefore, it remained unidentified for about 130 years ( Komposch, 1998), having been overlooked in many earlier reports from high-altitude localities. However, the presence of distinct dark horizontal markings on the leg coxae allows straightforward and reliable differentiation from *L. rupestre.*

**Figure 1.**
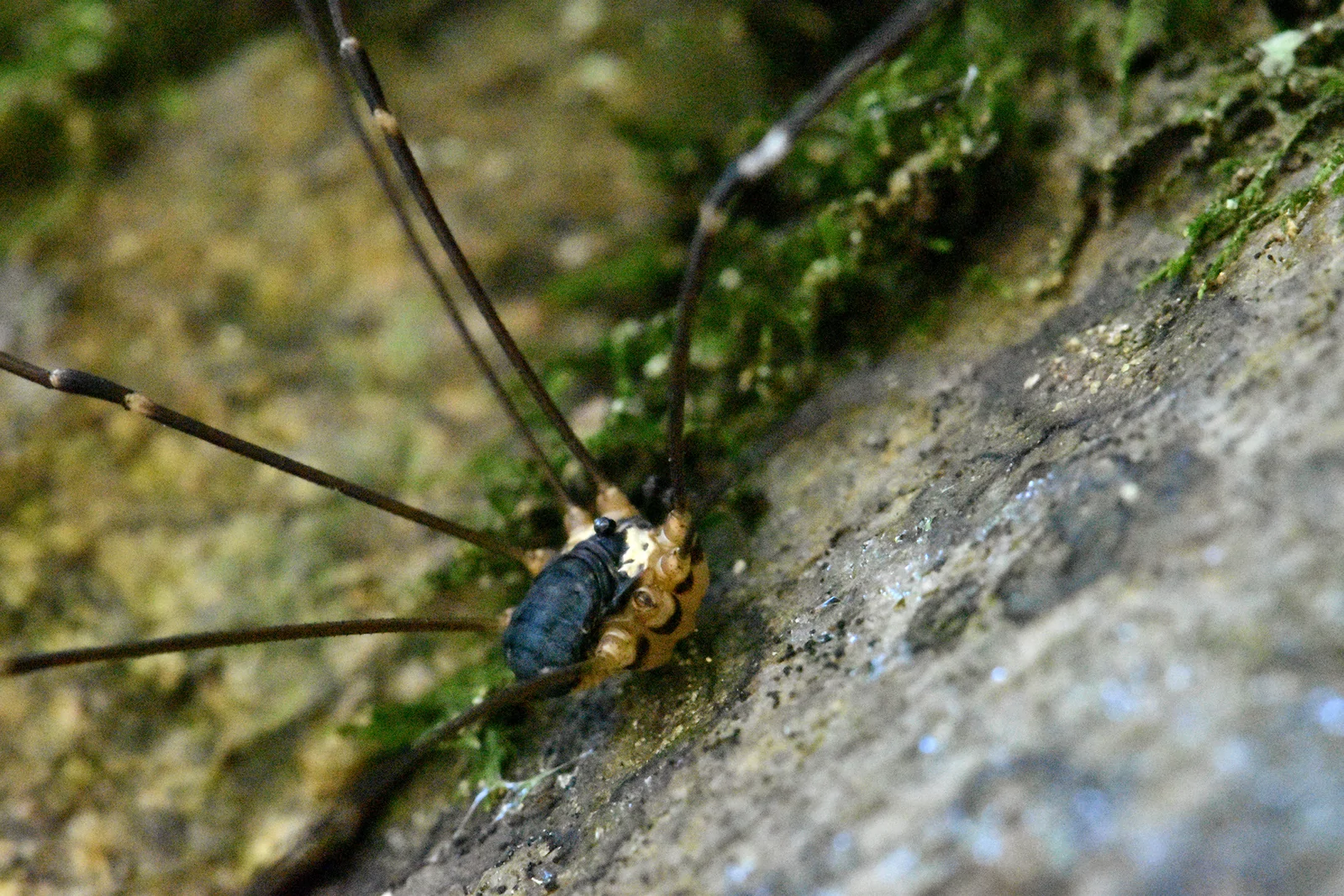
Male *L. subalpinum* in a typical rocky microhabitat in the Glažuta gorge, Slovenia.

*Leiobunum subalpinum* is currently not included in the IUCN Red List. However, it is listed in the national red lists of both Austria and Slovenia, where the species occurs. In Austria, it is classified as vulnerable on the Red List of Endangered Harvestmen and Spiders of Austria ( Komposch, 2009). In Slovenia, the species is classified as a rare species. This category includes species that are potentially endangered due to their rarity within the territory of Slovenia and that may quickly shift into the vulnerable category if additional threats arise ( PISRS, 2025).

Apart from a preliminary study by Kozel et al. (2021), there is little evidence from species-specific research explicitly analyzing the ecosystem function or ecological importance of *L. subalpinum.* Harvestmen are generally omnivorous and feed on a variety of food, including live prey they capture themselves, dead invertebrates, prey remains left by spiders, faeces from various animals, fungi, ripe fruits, and some plant juices, mostly from chewed material ( Martens, 1978; Acosta and Machado, 2007; Wijnhoven, 2011). Since *L. subalpinum* prefers cool, humid, rocky habitats and is sensitive to microclimatic variation, it may serve as important indicator species of montane–subalpine ecosystems ( Komposch, 1998; Kozel et al., 2021; Martens & Schönhofer, 2016). As the species is endemic to the Eastern Alps and restricted to a narrow geographical and altitudinal range, it represents an important regional biodiversity element and is therefore relevant for prioritizing the conservation of high-altitude habitats.

Since *L. subalpinum* is very similar to *L. rupestre* in most species-defining characters, a high-quality reference genome is essential for resolving genetic differences, investigating potential speciation between the two species, and advancing our understanding of its unique genetic makeup and adaptive traits.

The generation of this reference resource was coordinated by the European Reference Genome Atlas (ERGA) initiative’s Biodiversity Genomics Europe (BGE) project, supporting ERGA’s aims of promoting transnational cooperation to promote advances in the application of genomics technologies to protect and restore biodiversity ( Mazzoni et al., 2023).

## Materials & Methods

ERGA's sequencing strategy includes Oxford Nanopore Technology (ONT) and/or Pacific Biosciences (PacBio) for long-read sequencing, along with Hi-C sequencing for chromosomal architecture, Illumina Paired-End (PE) for polishing (i.e. recommended for ONT-only assemblies), and RNA sequencing for transcriptomic profiling, to facilitate genome assembly and annotation.

### Sample and sampling information

On June 14, 2023, Peter Kozel collected 14 male specimens of *L. subalpinum*, which were identified based on morphology using Martens (1978) by Peter Kozel and by barcoding of cytochrome c oxidase subunit I (COI) by Elena Bužan and Aja Bončina. Sampling took place on Glažuta, Mislinjski graben, Slovenia, and was performed under permission issued by the Slovenian Environmental Agency. Specimens were hand collected and placed in glass jars in the field. In the laboratory, each specimen was snap-frozen and subsequently preserved at -80 °C until DNA extraction.

### Vouchering information

Physical reference materials for the here sequenced specimen have been deposited in the invertebrate collection of the Slovenian Museum of Natural History (zbirka nevretenčarjev) https://www.pms-lj.si/en/ under accession number PMSL-Invertebrata-1.

A whole organism is available from a proxy voucher at the Biobank of the Slovenian Museum of Natural History https://www.pms-lj.si/en/ under voucher ID PMS_TIS_29.

### Data availability

*Leiobunum subalpinum* and the related genomic study were assigned to Tree of Life ID (ToLID) 'qqLeiSuba9' and all sample, sequence, and assembly information are available under the umbrella BioProject PRJEB86343. The sample information is available at the following BioSample accessions: SAMEA115177240 and SAMEA115177248. The genome assembly is accessible from ENA under accession number GCA_965184155.1 and the annotated genome is available through the Ensembl webpage ( https://projects.ensembl.org/erga-bge/). Sequencing data produced as part of this project are available from ENA at the following accessions: ERX14063879, ERX14063880, ERX14063881, and ERX14063882. Documentation related to the genome assembly and curation can be found in the ERGA Assembly Report (EAR) document available at https://github.com/ERGA-consortium/EARs/tree/main/Assembly_Reports/Leiobunum_subalpinum/qqLeiSuba9. Further details and data about the project are hosted on the ERGA portal at https://portal.erga-biodiversity.eu/data_portal/3086104.

### Genetic information

The estimated genome size, based on ancestral taxa, is 1.10 Gb. This corresponds to a diploid genome with a haploid number of 10 chromosomes (2n = 20). All information for this species was retrieved from Genomes on a Tree ( Challis et al., 2023).

### DNA/RNA processing

DNA was extracted from the whole organism using the Blood & Cell Culture DNA Mini Kit (Qiagen) following the manufacturer’s instructions. DNA quantification was performed using a Qubit dsDNA BR Assay Kit (Thermo Fisher Scientific), and DNA integrity was assessed using a Genomic DNA 165 Kb Kit (Agilent) on the Femto Pulse system (Agilent). The DNA was stored at +4 °C until sequenced.

RNA was extracted from the whole organism using a RNeasy Mini Kit (Qiagen) according to the manufacturer’s instructions. RNA quantification was performed using the Qubit RNA BR kit, and RNA integrity was assessed using a Bioanalyzer 2100 system (Agilent) RNA 6000 Nano Kit (Agilent). The RNA was stored at -80 °C until sequenced.

### Library preparation and sequencing

For long-read whole genome sequencing, a library was prepared using the SQK-LSK114 Kit (Oxford Nanopore Technologies, ONT), which was then sequenced using a R10.4.1 flow cell on a PromethION 24 A Series instrument (ONT). A short-read whole-genome sequencing library was prepared using the KAPA Hyper Prep Kit (Roche). A Hi-C library was prepared from the whole organism using the Dovetail Omni-C Kit (Cantata Bio), followed by the KAPA Hyper Prep Kit for Illumina sequencing (Roche). The RNA library was prepared using the KAPA mRNA Hyper prep kit (Roche). All short-read libraries were sequenced on a NovaSeq 6000 instrument (2x150bp, Illumina). In total 115x Oxford Nanopore, 140x Illumina WGS shotgun, and 210x HiC data were sequenced to generate the assembly.

### Genome assembly methods

The genome was assembled using the CNAG CLAWS pipeline ( Gomez-Garrido, 2024). Briefly, reads were pre-processed for quality and length using Trim Galore v0.6.7 and Filtlong v0.2.1, and initial contigs were assembled using NextDenovo v2.5.0, followed by polishing of the assembled contigs using HyPo v1.0.3 ( Kundu et al., 2019), removal of retained haplotigs using purge-dups v1.2.6 ( Guan et al., 2020) and scaffolding with YaHS v1.2a ( Zhou et al., 2023). Sequences corresponding to Pseudomonadota, Chordata or Molluca were found in 22 scaffolds with BlobToolKit v0.6.0 ( Challis et al., 2020) and removed from the assembly. Finally, assembled scaffolds were curated via manual inspection using Pretext v0.2.5 ( Harry, 2022) with the Rapid Curation Toolkit ( https://gitlab.com/wtsi-grit/rapid-curation ) to remove any false joins and incorporate any sequences not automatically scaffolded into their respective locations in the chromosomal pseudomolecules (or super-scaffolds). Sex chromosomes could not be identified. Summary analysis of the released assembly was performed using the ERGA-BGE Genome Report ASM Galaxy workflow ( https://doi.org/10.48546/workflowhub.workflow.1103.2).

### Genome annotation methods

A gene set was generated using the Ensembl Gene Annotation system ( Aken et al., 2016), primarily by aligning publicly available short-read RNA-seq data from BioSample SAMEA115177248 to the genome. Gaps in the annotation were filled via protein-to-genome alignments of a select set of clade-specific proteins from UniProt ( Consortium, 2019) which had experimental evidence at the protein or transcript level. At each locus, data were aggregated and consolidated, prioritising models derived from RNA-seq data, resulting in a final set of gene models and associated non-redundant transcript sets. To distinguish true isoforms from fragments, the likelihood of each open reading frame (ORF) was evaluated against known metazoan proteins. Low-quality transcript models, such as those showing evidence of fragmented ORFs, were removed. In cases where RNA-seq data were fragmented or absent, homology data were prioritised, favouring longer transcripts with strong intron support from short-read data. The resulting gene models were classified into two categories: protein-coding, and long non-coding. Models that did not overlap protein-coding genes and were constructed from transcriptomic data were considered potential lncRNAs. Potential lncRNAs were further filtered to remove single-exon loci due to their unreliability. Putative miRNAs were predicted by performing a BLAST search of miRBase ( Kozomara et al., 2019) against the genome, followed by RNAfold analysis ( Gruber et al., 2008). Other small non-coding loci were identified by scanning the genome with Rfam ( Kalvari et al., 2018) and passing the results through Infernal ( Nawrocki & Eddy, 2013). Summary analysis of the released annotation was carried out using the ERGA-BGE Genome Report ANNOT Galaxy workflow ( 10.48546/workflowhub.workflow.1096.1).

## Results

### Genome assembly

The genome assembly has a total length of 578,300,861 bp in 44 scaffolds ( Figures 2 & 3), with a GC content of 36.4%. The assembly has a contig N50 of 5,243,763 bp and L50 of 34 and a scaffold N50 of 52,575,036 bp and L50 of 5. The assembly has a total of 221 gaps, totalling 44.2 kb in cumulative size. The single-copy gene content analysis using the Arthropoda database with BUSCO ( Manni et al., 2021) resulted in 97.8% completeness (97.2% single and 0.6% duplicated). 82.8% of reads k-mers were present in the assembly and the assembly has a base accuracy Quality Value (QV) of 42.8 as calculated by Merqury ( Rhie et al., 2020).

**Figure 2.**
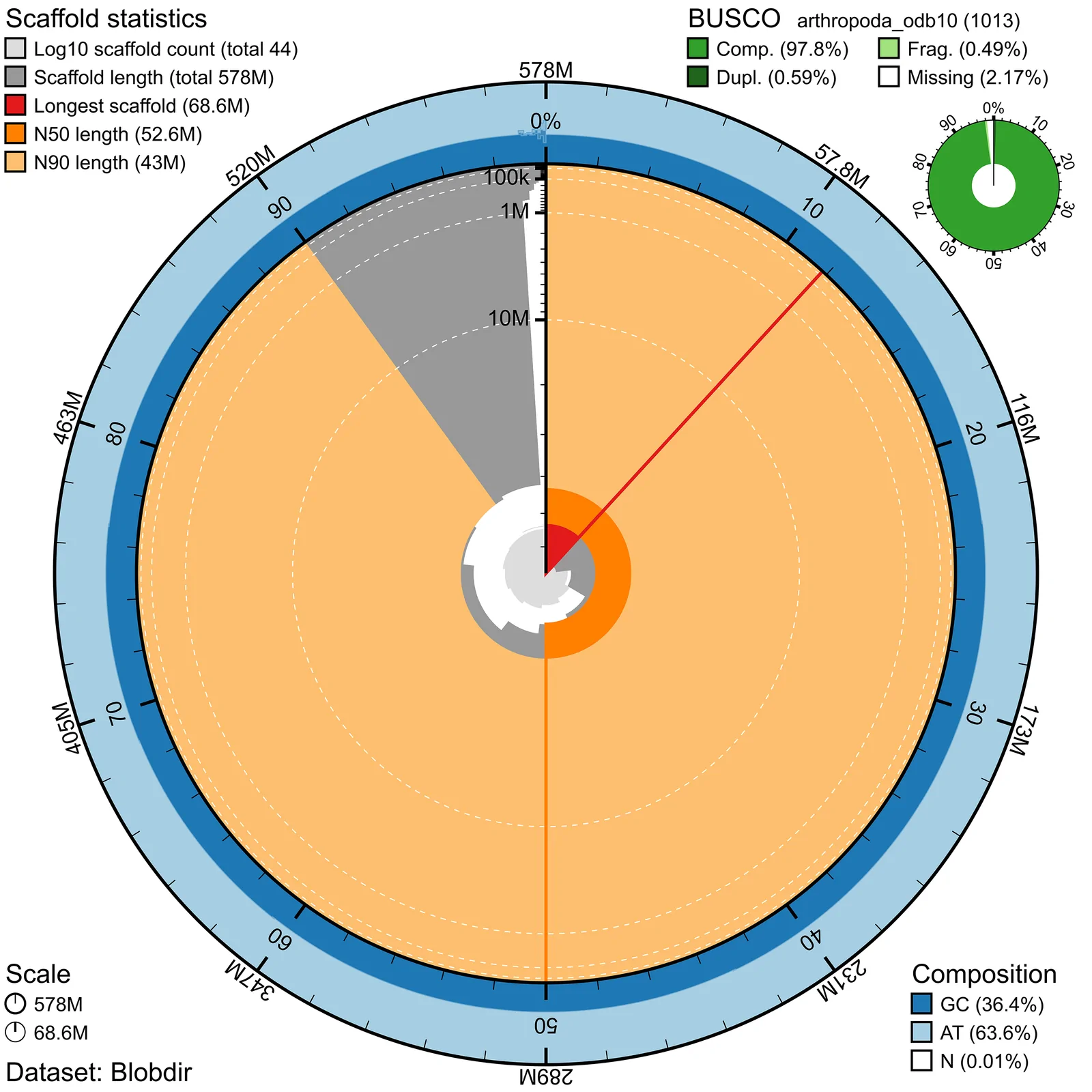
Snail plot summary of assembly statistics. The main plot is divided into 1,000 size-ordered bins around the circumference, with each bin representing 0.1% of the 578,300,861 bp assembly. The distribution of sequence lengths is shown in dark grey, with the plot radius scaled to the longest sequence present in the assembly (68.6 Mb, shown in red). Orange and pale-orange arcs show the scaffold N50 and N90 sequence lengths (52,575,036 and 43,002,158 bp), respectively. The pale grey spiral shows the cumulative sequence count on a log-scale, with white scale lines showing successive orders of magnitude. The blue and pale-blue area around the outside of the plot shows the distribution of GC, AT, and N percentages in the same bins as the inner plot. A summary of complete, fragmented, duplicated, and missing BUSCO genes found in the assembled genome from the Arthropoda database (odb10) is shown in the top right.

**Figure 3.**
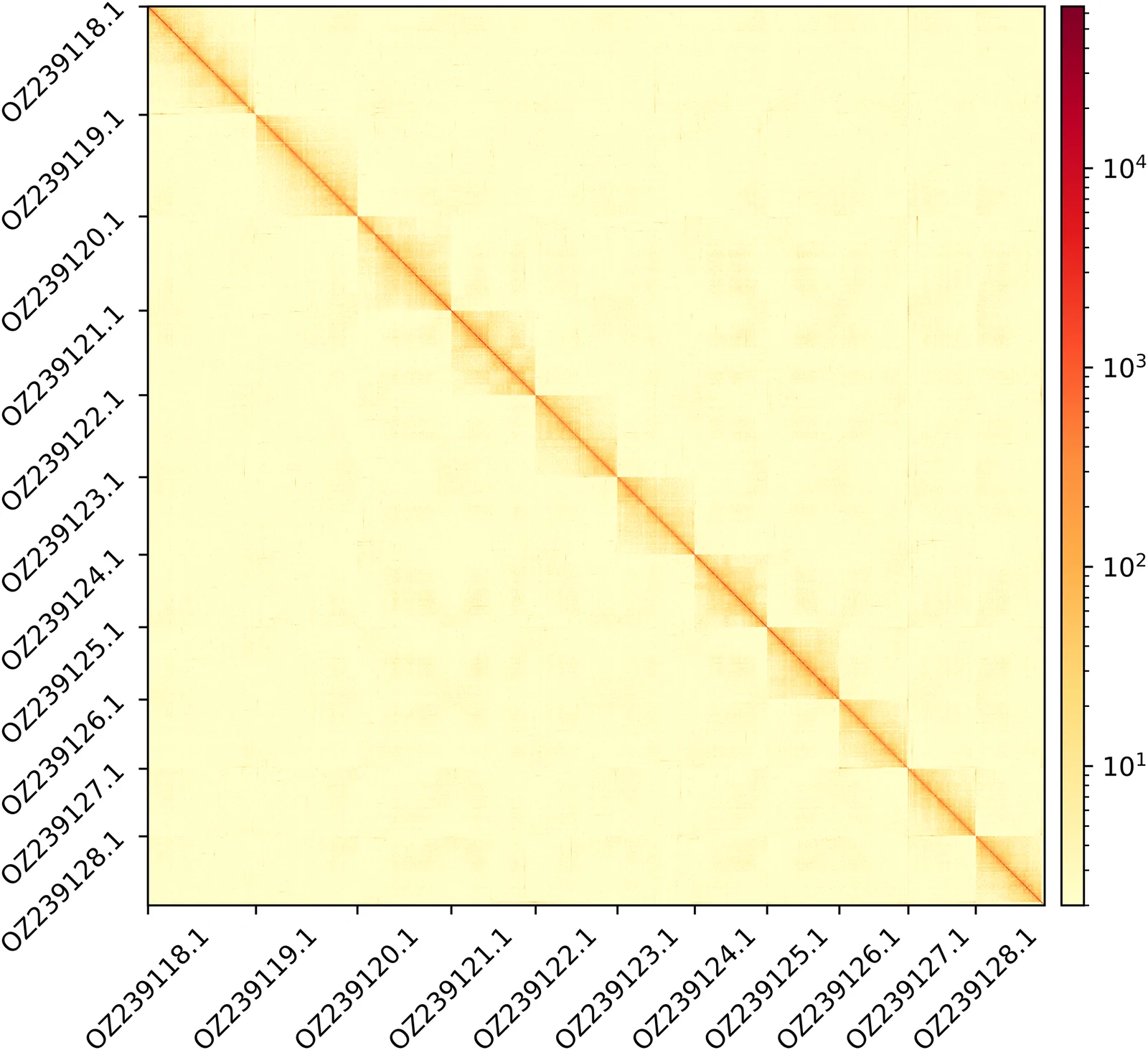
Hi-C contact map showing spatial interactions between regions of the genome. The diagonal corresponds to intra-chromosomal contacts, depicting chromosome boundaries. The frequency of contacts is shown on a logarithmic heatmap scale. Hi-C matrix bins were merged into a 100 kb bin size for plotting.

### Genome annotation

The genome annotation consists of 13,333 protein-coding genes with associated 23,640 transcripts, in addition to 21,543 non-coding genes ( Table 1). Using the longest isoform per transcript, the single-copy gene content analysis using the Arthropoda database with BUSCO resulted in 94.4% completeness. Using the Metazoa-v2.0.0.h5 database for OMArk ( Nevers et al., 2025) resulted in 90.5% completeness and 56.7% consistency ( Table 2).

**Table 1.** Statistics from assembled gene models.

	No. genes	No. transcripts	Mean gene length (bp)	No. single-exon genes	Mean exons per transcript
**mRNA**	13,333	23,640	10,765	337	8.1
**pseudogene**	0.0	0.0	0.0	0.0	0.0
**lncRNA**	3,831	4,294	4,118	24	2.3
**snoRNA**	20	20	171	20	1.0
**snRNA**	441	441	148	441	1.0
**rRNA**	1,632	1,632	154	1,632	1.0
**tRNA**	15,619	15,619	77	15,619	1.0
**Other ncRNA**	31,450	31,450	76-105	31,450	1.0-1.0

**Table 2.** Annotation completeness and consistency scores calculated by BUSCO run in protein mode (arthropoda_odb10) and OMArk (Metazoa-v2.0.0.h5).

	Complete	Singular	Duplicated	Fragmented	Missing
**BUSCO**	956 (94.4%)	948 (93.6%)	8 (0.8%)	7 (0.7%)	50 (4.9%)
**OMArk**	2,617 (90.5%)	2,517 (87.1%)	100 (3.4%)	-	274 (9.5%)

	Consistent	Inconsistent	Contaminants	Unknown
**OMArk**	7,565 (56.7%)	2,178 (16.3%)	0.0 (0.0%)	3,590 (26.9%)

## Author Contributions

EB and PK coordinated the project; PK collected the species; PK and AB identified the species; PK sampled and preserved biological material and provided metadata; AsB, RM, RF, and NE provided support in sampling, shipping of biological material, metadata collection, and management; MG and LA extracted DNA, prepared libraries, and performed sequencing; FCF, FC, and JG-G performed genome assembly and curation under the supervision of TSA; FM, VPBE, and LH performed genome annotation; CB generated the analysis and report. All authors contributed to the writing, review, and editing of this genome note and read and approved the final version.
