# Supplementary material for: ERGA-BGE genome of Leiobunum subalpinum - a montane-subalpine harvestman species endemic to the eastern Alps
Source: Open Res Eur. 2026 Mar 26;6:81. [Version 1] doi: 10.12688/openreseurope.21990.1 (PMC13662379; doi:10.12688/openreseurope.21990.1)
Supplement: Peer review history

# Reviewer report: Reviewer response for version 1

**DOI:** 10.21956/openreseurope.23794.r82186

**Published:** 2026-10-13

**Authors:** Craig Wilding

**Affiliations:** 1 University College Dublin, Belfield, Ireland

Kozel *et al*. present a high-quality assembly from montane Opilione native to the Slovenian/Austrian Alps. This assembly is small but of high quality, with impressive BUSCO scores (approximately 95% completeness). There is an excellent justification for this study, and I find no problems with the methodology. However, there are some details that could be clearer (see below).

 Whilst it is not critical, it would be good to be absolutely clear about which and how many specimens were used. The data availability section states: " *Leiobunum subalpinum* and the related genomic study were assigned to Tree of Life ID (ToLID) 'qqLeiSuba9' and all sample, sequence, and assembly information are available under the umbrella BioProject PRJEB86343. The sample information is available at the following BioSample accessions: SAMEA115177240 and SAMEA115177248." Whilst SAMEA115177240 relates to ToLID 'qqLeiSuba9', SAMEA115177248 relates to ToLID 'qqLeiSuba1'. Can the authors therefore clarify how many of the 14 samples collected were actually sequenced, and which specimens were used for each methodology? My reading of the results is that qqLeiSuba9 was used for genome sequencing and qqLeiSuba1 for RNA-Seq. Is this correct?

 Related to this, both BioSample accessions, SAMEA115177240 and SAMEA115177248, state 'not applicable' for sex. Surely sex is applicable here, and it should be relatively straightforward to establish the sex of the specimens sampled?

 The abstract states that "the genome sequence was assembled into 11 contiguous chromosomal pseudomolecules", but this is not explicitly mentioned in the results, where only the number of scaffolds is given. It is, of course, evident from Figure 3, but this could (and probably should) be stated explicitly, perhaps in the Genome Assembly section of the results.

 Gene annotation: my reading of the methodology is that gene annotation relied primarily on RNA-Seq data from qqLeiSuba1, supplemented by protein-to-genome alignments of a select set of clade-specific proteins from UniProt.

 My understanding is that, within the Darwin Tree of Life (DToL) pipeline, RNA-Seq evidence would typically be complemented by other approaches, such as *ab initio* gene prediction and broader homology-based evidence. Given that the RNA-Seq 

 data appear to come from a single, presumably adult specimen, I wonder whether genes not expressed at this life stage or in this sex might have been 

 missed? Whilst the high BUSCO completeness of the annotation is reassuring, BUSCO assesses the presence of a conserved set of genes and does not 

 necessarily therefore reflect the completeness of the annotation across different life stages or sexes. Could the authors clarify whether additional gene prediction approaches were used, and, if not, whether these were considered?

Are sufficient details of methods and materials provided to allow replication by others?

Yes

Is the rationale for creating the dataset(s) clearly described?

Yes

Are the datasets clearly presented in a useable and accessible format?

Yes

Are the protocols appropriate and is the work technically sound?

Yes

Reviewer Expertise:

Evolutionary genetics

I confirm that I have read this submission and believe that I have an appropriate level of expertise to confirm that it is of an acceptable scientific standard.

# Reviewer report: Reviewer response for version 1

**DOI:** 10.21956/openreseurope.23794.r82195

**Published:** 2026-10-10

**Authors:** Nikolaos Papadopoulos

**Affiliations:** 1 University of Vienna, Vienna, Austria

Overall, this is a very complete data report for a novel genome assembly. The raw data and the annotated assembly are already publicly available, which is exactly as it should be. The authors have used up-to-date open-source bioinformatic tools, for which I commend them.

 I only have a couple of minor issues:

 First, it seems important to mention the providence of the sequencing data without having to click through the ENA records. How many animals were used for short read DNA? How many for long read DNA? How many for HiC? How many for transcriptomics? Was whole organism was used in all cases? Perhaps most critically, was the assembly created on data from a single specimen?

 Second, the authors report on the use of various tools and pipelines for genome assembly and annotation. Do all steps refer to running methods on default parameters? If not, I would encourage the authors to publish config files or scripts in a small GitHub repository (or equivalent).

 Third, the authors correctly mention that they used BioSample SAMEA115177248 to predict gene models. It would be a courtesy to the reader to mention here that this is a BioSample from the same species and locality, in fact one that was generated by this exact study, instead of pointing to an already "publicly available RNA-seq" dataset.

 Fourth, which clade-specific proteins were picked out of UniProt, and why? While I am not doubting the validity or intention of the authors' approach, it would be nice to have slightly more detail (or indeed a way to reproduce their analysis, instead of relying solely on good faith). Overall, I would appreciate some hard documentation for the genome annotation methods. Again, a config file would suffice, since the Ensembl Gene Annotation pipeline is publicly available.

 The issues mentioned here should be easily and quickly addressable, and do not detract considerably from what is overall a very good genome data note.

Are sufficient details of methods and materials provided to allow replication by others?

Partly

Is the rationale for creating the dataset(s) clearly described?

Yes

Are the datasets clearly presented in a useable and accessible format?

Yes

Are the protocols appropriate and is the work technically sound?

Yes

Reviewer Expertise:

genomics and transcriptomics of non-model species; evo-devo; single-cell transcriptomics; statistical modelling

I confirm that I have read this submission and believe that I have an appropriate level of expertise to confirm that it is of an acceptable scientific standard.
